# Humoral and Cellular Immunogenicity and Safety of Five Different SARS-CoV-2 Vaccines in Patients With Autoimmune Rheumatic and Musculoskeletal Diseases in Remission or With Low Disease Activity and in Healthy Controls: A Single Center Study

**DOI:** 10.3389/fimmu.2022.846248

**Published:** 2022-03-31

**Authors:** Gábor J. Szebeni, Nikolett Gémes, Dániel Honfi, Enikő Szabó, Patrícia Neuperger, József Á. Balog, Lajos I. Nagy, Zoltán Szekanecz, László G. Puskás, Gergely Toldi, Attila Balog

**Affiliations:** ^1^ Biological Research Centre, Laboratory of Functional Genomics, Szeged, Hungary; ^2^ Department of Physiology, Anatomy and Neuroscience, Faculty of Science and Informatics, University of Szeged, Szeged, Hungary; ^3^ CS-Smartlab Devices, Kozarmisleny, Hungary; ^4^ Doctoral School in Biology, Faculty of Science and Informatics, University of Szeged, Szeged, Hungary; ^5^ Department of Rheumatology and Immunology, Faculty of Medicine, Albert Szent-Gyorgyi Health Centre, University of Szeged, Szeged, Hungary; ^6^ Avidin Ltd., Szeged, Hungary; ^7^ Division of Rheumatology, Faculty of Medicine, University of Debrecen, Debrecen, Hungary; ^8^ Liggins Institute, University of Auckland, Auckland, New Zealand

**Keywords:** SARS-CoV-2 vaccination, rheumatic and musculoskeletal diseases, anti-RBD neutralizing antibodies, CD4^+^ T-cell response, CD8^+^ T-cell response

## Abstract

**Background:**

Vaccine-induced immunity is essential for controlling the COVID-19 pandemic. Data on humoral and cellular immunogenicity and safety of different SARS-CoV-2 vaccines in patients with autoimmune rheumatic and musculoskeletal diseases (RMDs) are limited.

**Methods:**

A single center observational study evaluated the immunogenicity and safety of the two-dose regimen of the BBIBP-CorV inactivated, Gam-COVID-Vac and AZD1222 adenovirus-based, and BNT162b2 and mRNA-1273 mRNA-based vaccines in patients with RMDs (n = 89) compared with healthy controls (n = 74). Neutralizing anti-RBD (receptor binding domain) specific antibodies and SARS-CoV-2 specific T-cell response were measured one and four months after the second vaccine dose in parallel with vaccination efficacy and safety.

**Results:**

Disease-specific comparison showed that antibody response at four months was higher in spondylarthropathies compared to rheumatoid arthritis and autoimmune RMDs. Risk factors for reduced immunogenicity included longer disease duration, positive immunoserological profile and anti-CD20 therapy of patients. The rate of positive anti-RBD antibody response for healthy controls versus patients after 4 months post vaccination was 69% vs. 55% for the inactivated viral vaccine BBIBP-CorV, 97% vs. 53% for the pooled data of adenovirus vector-based vaccines Gam-COVID-Vac and AZD1222, or 100% vs. 81% for the pooled data of mRNA vaccines BNT162b2 and mRNA-1273, respectively. Patients who received the Gam-COVID-Vac or mRNA-1273 vaccines had a higher proportion of TNF-α producing CD4+ T-cells upon SARS-CoV-2 antigen stimulation compared to the inactivated viral vaccine.

**Conclusion:**

All five investigated vaccines were immunogenic in the majority of patients and healthy controls with variable antibody and T-cell response and an acceptable safety profile.

## Introduction

Efficient control of the COVID-19 pandemic has become a crucial public health and economic priority worldwide. Vaccination against the SARS-CoV-2 virus has proven to be a cornerstone of preventative strategies. The BBIBP-CorV inactivated viral, Gam-COVID-Vac and AZD1222 adenovirus-based, and BNT162b2 and mRNA-1273 mRNA-based vaccines have demonstrated a high efficacy rate with an acceptable safety profile (1–5).

Patients with autoimmune rheumatic and musculoskeletal diseases (RMDs) are at increased risk of infections, including vaccine-preventable infectious diseases (6). While vaccinations are essential in their management, the drugs used to treat RMDs may reduce responses to vaccines. Available data regarding the effect of disease modifying anti-rheumatic drugs (DMARDs) on vaccine immunogenicity, and vaccination recommendations for RMD patients were summarized recently (7). An active disease with ongoing inflammatory response is known to be associated with a higher risk of infections and reduced response to vaccination (8, 9). However, the effect of vaccination in patients with low disease activity and stable immune suppressive therapy is less described. In particular, the effect of disease duration on the immunogenicity of SARS-CoV-2 vaccines has only been partially investigated. Another question of interest is whether patients receiving biological disease modifying anti-rheumatic drug (bDMARD) therapy, and specifically those on B-cell inhibitors, show a reduced response to SARS-CoV-2 vaccination (10, 11).

Prioritized vaccination of patients with autoimmune and inflammatory RMDs to reduce COVID-19 risk was proposed by the American College of Rheumatology (ACR) (12), and a considerable amount of data on the efficacy and safety of mRNA-based vaccination in immunosuppressed patients is available (11). However, data on adenovirus-based and inactivated SARS-CoV-2 vaccines in patients with autoimmune and inflammatory RMDs are limited (13, 14).

Therefore, we aimed to perform a prospective observational study to evaluate the humoral and cellular immunogenicity, efficacy, and safety of the BBIBP-CorV inactivated viral, Gam-COVID-Vac and AZD1222 adenovirus-based, and BNT162b2 and mRNA-1273 mRNA-based vaccines in patients with autoimmune and inflammatory RMDs compared with healthy controls.

## Materials and Methods

### Ethics Statement

The study involving human participants was reviewed and approved by the Human Investigation Review Board of the University of Szeged under the Project Identification Code 96/2021-SZTE-KREB. The patients/participants provided their written informed consent to participate in this study. Healthy controls were staff members of the Biological Research Centre of Szeged, Hungary or the University of Szeged, Hungary. Subjects were informed about the study by a physician and acute SARS-CoV-2 infection was ruled out by qPCR. Laboratory studies and interpretations were performed on coded samples lacking personal and diagnostic identifiers. The study was adhered to the tenets of the most recent revision of the Declaration of Helsinki.

### Study Design

This prospective observational study was conducted at the Department of Rheumatology and Immunology of Albert Szent-Györgyi Medical School, University of Szeged, Hungary between May 2021 and September 2021. The primary end point was the humoral and cellular immunogenicity of the BBIBP-CorV inactivated viral, Gam-COVID-Vac and AZD1222 adenovirus-based, and BNT162b2 and mRNA-1273 mRNA-based vaccines in adult patients with autoimmune and inflammatory RMDs compared with healthy controls measured at one and four months following the second vaccine dose. Secondary end points included:

1. Effect of immunosuppressive treatment on the production of anti-RBD neutralizing antibodies and SARS-CoV-2 specific T-cell response in RMD patients.
2. Comparison of the production of anti-RBD neutralizing antibodies and SARS-CoV-2 specific T-cell response based on the type of vaccination.

### Study Population

Characteristics of the study participants (89 patients with autoimmune and inflammatory RMDs and 74 healthy controls) and the type of SARS-CoV-2 vaccination they received are summarized in **Table 1**. All participants received two doses of the relevant vaccine in line with recommendations of the respective manufacturer (BBIBP-CorV—Beijing Institute, China; Gam-COVID-Vac—Gamaleya Research Institute, Russia; AZD1222—AstraZeneca, UK; BNT162b2—Pfizer-BioNTech, USA; and mRNA-1273—Moderna, USA). Second shots were administered 4 weeks after the first dose for all types of the investigated vaccines. Adult patients were enrolled during regular visits at the Department of Rheumatology and Immunology (University of Szeged, Hungary) based on the following inclusion criteria: rheumatoid arthritis (RA)—ACR/European League Against Rheumatism (EULAR) 2010 classification criteria (15); psoriatic arthritis (PsA)—Classification Criteria for PsA (16); axial spondyloarthritis (axSpA)—Assessment of SpondyloArthritis International Society classification criteria (17); systemic lupus erythematosus (SLE)—ACR 1997 (18) or Systemic Lupus Erythematosus International Collaborating Clinics 2012 criteria (19); systemic vasculitis, namely, large vessel vasculitis (LVV), antineutrophil cytoplasmic antibody-associated vasculitis (AAV), granulomatosis with polyangiitis (GPA) and eosinophilic GPA—Chapel Hill Consensus Conference definitions (20); Sjögren syndrome (SS) ACR/EULAR 2016 criteria (21); systemic sclerosis (SSc)—ACR/EULAR 2013 criteria (22); idiopathic inflammatory myopathy (IIM)—ACR/EULAR 2017 classification criteria (23); Behcet’s disease—International Criteria for Behcet’s Disease (ICBD) (24). All patients were either in remission or had a low disease activity. All patients were on stable medication for at least the last eight weeks before enrolment. A stable dose of glucocorticoid (GC) ≤4 mg per day was permitted. The medication of the patients is summarized in **Table 2**. Patients were instructed to continue all medications during the vaccination period, except for rituximab treatment which was delayed after the vaccination. Exclusion criteria for all groups were previous COVID-19 infection, pregnancy and history of past vaccination allergy. Additional exclusion criteria for controls were history of RMDs and immunosuppressive treatment.

**Table 1:** Demographic characteristics and the type of SARS-CoV-2 vaccination of patients with autoimmune and inflammatory RMDs and healthy controls.

	Age, years	Female	Disease duration, years	BBIBP-CorV	Gam-Covid-Vac	AZD1222	BNT162b2	mRNA-1273
Controls, n = 74	43 ± 12.5	46 (62%)	–	18 (24%)	16 (22%)	14 (19%)	16 (22%)	10 (13%)
All patients with aiRMDs, n = 89	59 ± 14	64 (72%)	11 ± 8.4	11 (12%)	8 (9%)	20 (22%)	34 (39%)	16 (18%)
RA, n = 41	62 ± 10.1	33 (83%)	12 ± 9.1	6 (15%)	5 (12%)	8 (19%)	20 (49%)	2 (5%)
PsA, n = 7	50 ± 6.6	4 (57%)	9 ± 6.6	0	1 (14%)	1 (14%)	2 (29%)	3 (43%)
AxSpa, n = 12	48 ± 14.1	1 (8%)	13 ± 10.2	2 (17%)	0	1 (8%)	4 (33%)	5 (42%)
SLE n = 11	52 ± 14.1	1 (9%)	13 ± 9.4	0	1 (9%)	3 (27%)	4 (37%)	3 (27%)
SS n = 4	62 ± 12	4 (100%)	9 ± 3.9	1 (25%)	0	2 (50%)	1 (25%)	0
IIM n= 1	75	1 (100%)	6	0	0	0	1 (100%)	0
SSc n = 1	73	1 (100%)	3	0	0	0	1 (100%)	0
LVV, n = 5	70 ± 6.3	4 (80%)	4 ± 3.3	1 (20%)	0	0	2 (40%)	2 (40%)
AAV, n = 6	69 ± 11	4 (67%)	8 ± 2.9	0	1 (17%)	1 (17%)	3 (50%)	1 (17%)
Other vasculitis, n = 1	54	1 (100%)	13	0	0	1 (100%)	0	0

Data are presented as mean ± SD or n (%). AAV, antineutrophil cytoplasmic antibody (ANCA)-associated vasculitis; aiRMD, autoimmune and inflammatory rheumatic and musculoskeletal disease; AxSpA, axial spondyloarthritis; IIM, idiopathic inflammatory myositis; LVV, large vessel vasculitis; PsA, psoriatic arthritis; RA, rheumatoid arthritis; SLE, systemic lupus erythematosus, SS, Sjögren syndrome; SSc, systemic sclerosis.

**Table 2:** Therapies used in patients with autoimmune and inflammatory RMDs.

	GC	cDMARD	bDMARD	Anti-CD20	BLySi	JAKi
All patients with aiRMDs, n = 89	16 (18%)	43 (48%)	35 (39%)	8 (9%)	2 (2%)	1 (1%)
RA, n = 41	3 (7%)	17 (41%)	18 (44%)	4 (10%)	0	1 (2%)
PsA, n = 7	0	2 (29%)	5 (71%)	0	0	0
AxSpa, n = 12	0	1 (8%)	11 (92%)	0	0	0
SLE n = 11	4 (36%)	9 (8%)	0	0	2 (18%)	0
SS n = 4	1 (25%)	4 (100%)	0	0	0	0
IIM n= 1	1 (100%)	1 (100%)	0	1 (100%)	0	0
SSc n = 1	0	0	0	1 (100%)	0	0
LVV, n = 5	3 (60%)	3 (60%)	1 (20%)	0	0	0
AAV, n = 6	3 (50%)	5 (83%)	0	2 (33%)	0	0
Other vasculitis, n = 1	1 (100%)	1 (100%)	0	0	0	0

Data are presented as n (%). AAV, antineutrophil cytoplasmic antibody (ANCA)-associated vasculitis; aiRMD, autoimmune and inflammatory rheumatic and musculoskeletal disease; AxSpA, axial spondyloarthritis; IIM, idiopathic inflammatory myositis; LVV, large vessel vasculitis; PsA, psoriatic arthritis; RA, rheumatoid arthritis; SLE, systemic lupus erythematosus, SS, Sjögren syndrome; SSc, systemic sclerosis; GC, glucocorticoids; cDMARD, conventional DMARD, namely, methotrexate, leflunomide, azathioprine, chloroquine, hidroxychloroquin; bDMARD, biological DMARD, namely, tumor necrosis factor alpha inhibitors (12 patients with RA and 8 patients with AxSpa and 2 patients with PsA), interleukin 17 inhibitors (3 patients with AxSpa and 3 patients PsA), interleukin 6 inhibitors (6 patients with RA and 1 patients with LVV); LySi, BLyS-specific inhibitor, belimumab; JAKi, janus kinase inhibitor.

### Measurement of Neutralizing Anti-RBD Specific Antibodies

Peripheral venous blood was taken into serum separator tubes (BD Vacutainer, Becton Dickinson, Sunnyvale, CA, USA). Sera were separated by centrifugation at 1,000*g* for 15 min. Quantitative measurement of neutralizing anti-RDB specific IgG-type antibody titers was performed with the Siemens Advia Centaur XPT system using the Siemens Healthineers SARS-CoV-2 IgG assay (sCOVG) (Siemens Healthineers, Munich, Germany). This is a fully automated two−step sandwich immunoassay using indirect chemiluminescent technology. Briefly, sera were incubated with the Solid Phase Reagent containing a preformed complex of streptavidin-coated microparticles and biotinylated SARS-CoV-2 recombinant antigens (S1 RBD, Wuhan strain) capturing SARS-CoV-2 specific antibodies in the specimen. The antibody–antigen complex was washed, and Lite Reagent was added consisting of an acridinium-ester-labeled anti-human IgG mouse monoclonal antibody. The entire complex was washed, and the signal was generated in the presence of Lite Reagent bound to the Solid Phase *via* the anti-SARS-CoV-2 IgG: SARS-CoV-2 antigen complex. When a direct relationship exists between the amount of SARS-CoV-2 IgG antibody present in the sample, the amount of relative light units (RLUs) is detected by the system. A reactive or non-reactive result was determined by the sCOVG Index Value established with the calibrators. The analytical measurement interval was 0.50–150.00 Index (U/ml). Non-reactive: <1.0 Index; the sample was considered negative for SARS-CoV-2 antibodies. Reactive: ≥1.0 Index; the sample was considered positive for SARS-CoV-2 antibodies. Measured Index Values were converted into WHO 20/136 approved international units of 1,000 Binding Antibody Unit per milliliter (BAU/ml) using the following equation: (sCOVG Index) ∗ 21.8 = 1 BAU/ml, where the diagnostic cut-off value was 21.8 BAU/ml) (25).

### Measurement of SARS-CoV-2 Specific T-Cell Response

Peripheral venous blood was taken into Lithium Heparin treated tubes (BD Vacutainer, Becton Dickinson, Sunnyvale, CA, USA). Peripheral blood mononuclear cells (PBMCs) were isolated by Ficoll density gradient centrifugation using Leucosep tubes (Greiner Bio-One, Kremsmünster, Austria). Cells were pelleted by centrifugation at 800*g* for 20 min. The ring of PBMCs was harvested by pipetting and diluted with 15 ml PBS, then centrifuged at 350*g* for 5 min. The supernatant (S/N) was removed. If necessary, red blood cells were lysed by 2 ml ACK solution (prepared in our laboratory: 0.15 M NaH_4_Cl, 10 mM KHCO_3_, 0.1 mM Na_2_EDTA, pH7.4, Merck, USA) at room temperature (RT) for 2 min. Cells were washed with 15 ml PBS and centrifuged at 350*g* for 5 min. Cells were resuspended in 320 µl complete RPMI-1640 cell culture media (Lonza, Switzerland) containing 10% FCS (Euroclone, Italy), 100 U/ml penicillin sodium salt and 100 µg/ml streptomycin sulfate salt (Merck, USA). PBMCs were divided into 3 wells for the following samples (1): untreated, (2) S-M-N Peptivator (Miltenyi Biotec, Germany) stimulated, (3) polyclonal activator stimulated (Cytostim, Miltenyi Biotec, Germany) in 100 µl/well on a 96-well plate (Corning, USA).

The SARS-CoV-2-specific T-cell response was measured using the SARS-CoV-2 T-Cell Analysis Kit for human PBMCs according to the instructions of the manufacturer (Miltenyi Biotec, Germany) with the following peptide pools: Peptivator SARS-CoV-2 Prot_M 6 nmol/peptide, Peptivator SARS-CoV-2 Prot_N 6 nmol/peptide, Peptivator SARS-CoV-2 Prot_S 6nmol/peptide. Peptide pools were dissolved in 200–200 µl sterile water/10% DMSO. Then 2-2 µl of S, M, and N peptide pools were added to the S-M-N stimulated wells, while 2 µl CytoStim was added to the polyclonal activator stimulated wells. After incubation at 37°C, 5% CO2 for 2h, Brefeldin-A was added to all wells at 2 µg/ml final concentration. Cells were incubated at 37°C, 5% CO2 for 16 h. Next day 100 µl cold (4°C) PEB buffer (0.5% BSA, 2 mM EDTA in PBS) was added to the wells. Cells were resuspended and pipetted into 12 × 75 mm FACS tubes (VWR International, USA) and diluted with 500 µl PEB buffer. Cells were centrifuged at 500*g* for 5 min, the S/N was removed. The Viobility 405/452 Fixable Dye was dissolved in 100 µl DMSO, then the working concentration of 100× dilution was prepared in PBS. Cells were resuspended in 100 µl Viobility Fixable Dye and incubated at RT for 10 min. Cells were washed with 500 µl PBS, centrifuged at 500*g* for 5 min. The S/N was removed, cells were resuspended in 100 µl PEB buffer. Approximately 100 µl Inside Fix solution was added to each sample and incubated at RT for 20 min. Cells were washed with 500 µl PBS, centrifuged at 500*g* for 5 min. The S/N was removed, then 100 µl Inside Perm was added to each sample. Cells were resuspended, incubated for 3 min at RT, diluted with 600 µl PBS, then centrifuged at 500*g* for 5 min. The S/N was removed then the antibody cocktail was added to the cells in 100 µl Inside Perm. The antibody cocktail consisted of 100× dilutions of anti-CD3 APC (clone REA613), anti-CD4 VioBright B515 (clone REA623), anti-CD8 VioGreen (clone REA734), anti-IFN-γ PE (clone 45-15), anti-TNF-α PE-Vio770 (clone cA2), anti-CD14 VioBlue (clone TÜK4), antiCD20 VioBlue (clone LT20), and anti-CD154 (CD40L) APC-Vio770. Cells were incubated at RT for 30 min. Cells were washed with 500 µl PBS, centrifuged at 500*g* for 5 min. Cells were resuspended in 400 µl PEB and minimum 1 × 10^5^ CD3^+^ cells were acquired on Cytoflex S fluorescence activated cell sorter (FACS) (Beckman Coulter, USA). Manual gating was used to determine CD4^+^ or CD8^+^ T-cells within live CD14^−^ CD20^−^ CD3^+^ lymphocytes in CytExpert (Beckman Coulter) (**Supplementary Figure 1A**). Reactive cells were gated as CD4^+^TNF-α^+^, CD4^+^IFNγ^+^, CD4^+^CD40L^+^, CD8^+^TNF-α^+^ and CD8^+^IFNγ^+^ upon S-M-N or polyclonal stimuli (**Supplementary Figure 1B**). Cell numbers in the reporting gates were normalized to parental CD4^+^ or CD8^+^ cells (reactive cell number/parental cell number × 10^6^), then background was normalized *via* subtraction of untreated from the stimulated. Finally, reactive cell numbers are shown in relation to 10^6^ CD4^+^ or CD8^+^ T-cells, the diagnostic cut-off value was 400 reactive cells of 10^6^ parental population.

### Statistical Analysis

Data are expressed as median [interquartile range]. Comparisons were made using Kruskal–Wallis, Mann–Whitney and Wilcoxon signed rank tests, as data were non-normally distributed according to the Kolmogorov–Smirnov test. p-values less than 0.05 were considered significant. Statistics were calculated using the GraphPad Prism 8 software (GraphPad, San Diego, CA, USA).

## Results

### Clinical Characteristics

There was no significant flare or relapse among patients with autoimmune and inflammatory RMDs during the observation period following vaccination. No alteration or adjustment of DMARD therapy or the dosage of GC following vaccination was necessary in any of the patients. None of the participants were diagnosed with COVID-19 infection. The safety profile of vaccines amongst healthy controls and patients is presented in **Supplementary Tables 1** and **2**, respectively.

### Humoral Immunogenicity of the Investigated Vaccines

**Figure 1:**
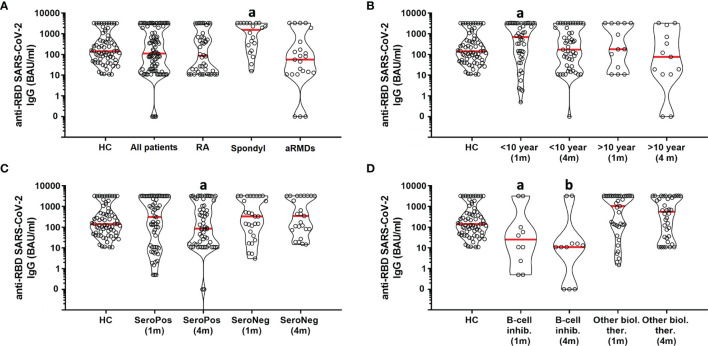
Influence of clinical parameters on SARS-CoV-2 neutralizing antibody levels in RMD patients. HC, healthy controls; RA, rheumatoid arthritis; Spondyl, spondylarthropathies; aRMDs, autoimmune rheumatic and musculoskeletal diseaases. **(A)** a vs. HC, All patients, RA, aRMDs (p = 0.0048). **(B)** a vs. HC, <10 year (4 m), >10 year (1 m), >10 year (4 m) (p = 0.0158). **(C)** a vs. SeroPos (1 m), SeroNeg (1 m), SeroNeg (4 m) (p = 0.0036). **(D)** a vs. Other biol. ther. (1 m) (p = 0.0074), b vs. Other biol. ther. (4 m) (p = 0.0055).

The antibody response was compared between RA, spondylarthropathies (axSpA and PsA) and autoimmune RMDs (SSc, SLE, SS, IIM, LVV, AAV, and Behcet disease) at one- and four-months post vaccination. The antibody level was higher at four months in spondylarthropathies compared to RA and autoimmune RMDs (**Figure 1A**, p = 0.0048). Following this, we compared patient subgroups based on disease duration (<10 years versus ≥10 years) and immune serological (autoantibody) profile. The anti-RBP antibody response was higher at one month compared to four months post vaccination in patients who were diagnosed <10 years ago (**Figure 1B**, p = 0.0158), whereas it was comparable in patients diagnosed ≥10 years ago. The antibody response was lower at four months compared to one month in seropositive patients (**Figure 1C**, p = 0.0036), whereas it was comparable in seronegative patients. We also compared patients receiving conventional DMARDs to those on biological DMARD therapy. Within the subgroup of patients on bDMARD therapy, those on B-cell inhibitory treatment showed lower antibody levels both at one- and four-months post vaccination compared to those receiving other biologicals (**Figure 1D**, p = 0.0074 and p = 0.0055, respectively).

**Figure 2:**
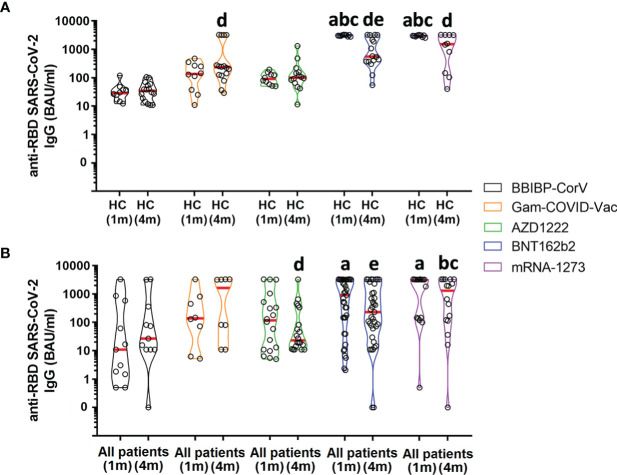
SARS-CoV-2 neutralizing antibody levels in healthy controls and RMD patients receiving different types of vaccines. HC, healthy controls. **(A)** a vs. BBIBP-CorV (1 m) (p <0.0001), b vs. Gam-COVID-Vac (1 m) (p <0.0001), c vs. AZD1222 (1 m) (p <0.0001), d vs. BBIBP-CorV (4 m) (p = 0.0106), e vs. BNT162b2 (1 m) (p <0.0001). **(B)** a vs. BBIBP-CorV (1 m) (p = 0.0122), b vs. BBIBP-CorV (4 m) (p = 0.0107), c vs. AZD1222 (4 m) (p = 0.0107), d vs. AZD1222 (1 m) (p = 0.0179), e vs. BNT162b2 (1 m) (p = 0.0298).

Next, we compared healthy controls and patients based on the vaccines they received. In healthy controls, vaccination with BNT162b2 or mRNA-1273 vaccines produced a higher antibody level at one month post vaccination in comparison to the other vaccines (**Figure 2A**, p <0.0001). Antibody levels decreased by 4 months compared to one month post vaccination in those who received the BNT162b2 vaccine (**Figure 2A**, p = 0.0106). However, antibody levels at four months were still higher in individuals vaccinated with Gam-COVID-Vac, BNT162b2 or mRNA-1273 vaccines compared to those who received the BBIBP-CorV vaccine (**Figure 2A**, p <0.0001). In patients, vaccination with BNT162b2 or mRNA-1273 vaccines produced a higher antibody level at one month post vaccination in comparison to the BBIBP-CorV vaccine (**Figure 2B**, p = 0.0122). Antibody levels at four months were higher in patients vaccinated with the mRNA-1273 vaccine compared to those who received BBIBP-CorV or AZD1222 vaccines (**Figure 2B**, p = 0.0107). Antibody levels decreased by 4 months compared to one month post vaccination in those who received AZD1222 (**Figure 2B**, p = 0.0179) or BNT162b2 vaccines (**Figure 2B**, p = 0.0298).

Data of AZD1222 and Gam-COVID-Vac adenovirus vector-based vaccines or BNT162b2 and mRNA-1273 mRNA vaccines were pooled to calculate the rate of individuals with a positive anti-RBD antibody response to the same type of vaccine at 4 months post vaccination. The satisfactory response rate for healthy controls versus patients with autoimmune and inflammatory RMDs was 69% vs. 55% for the inactivated viral vaccine (BBIBP-CorV), 97% vs. 53% for the adenovirus vector-based vaccines (Gam-COVID-Vac and AZD1222), and 100% vs. 81% for the mRNA vaccines (BNT162b2 and mRNA-1273), respectively.

### Cellular Immunogenicity of the Investigated Vaccines

No difference was detected in the studied cellular parameters when RA, spondylarthropathies and autoimmune RMDs were compared. Similarly, no difference was noted based on disease duration or biological therapy received. The proportion of TNF-α producing CD4 cells was higher in seronegative compared to seropositive patients upon stimulation with S/M/N-peptides (411 [188–670] vs 176 [0–466] cells/10^6^ reactive CD4 cell, p = 0.0112), whereas there was no difference upon polyclonal stimulation.

**Figure 3:**
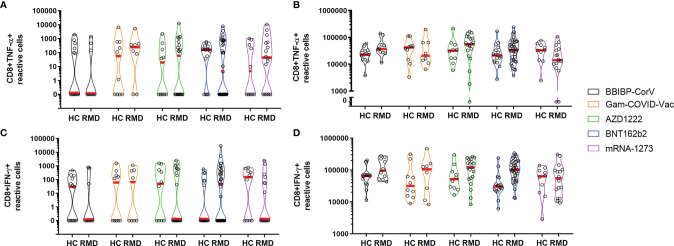
CD8^+^ T-cell responses in healthy controls and RMD patients receiving different types of vaccines upon S/M/N peptide **(A, C)** or polyclonal **(B, D)** stimulation. HC, healthy controls; RMD, rheumatic and musculoskeletal disease. No statistically significant difference was detected.

There was no statistically significant difference in the prevalence of reactive TNF-α- (**Figures 3A, B**) or IFN-γ-producing CD8^+^ T cells (**Figures 3C, D**) upon S/M/N or polyclonal stimulation between healthy controls or patients receiving different vaccine types. The proportion of CD8^+^ TNF-α^+^ responders amongst healthy controls versus patients 4 months post vaccination were 23% vs. 20% for BBIBP-CorV, 23% vs. 25% for pooled cases of Gam-COVID-Vac and AZD1222, and 15% vs 32% for pooled cases of BNT162b2 and mRNA-1273 vaccinated individuals. The percentage of CD8^+^ IFNγ^+^ responders among healthy controls versus patients 4 months post vaccination were 8% vs 20% for BBIBP-CorV, 23% vs 32% for pooled cases of Gam-COVID-Vac and AZD1222, and 15% vs 32% for pooled cases of BNT162b2 and mRNA-1273 vaccinated individuals.

**Figure 4:**
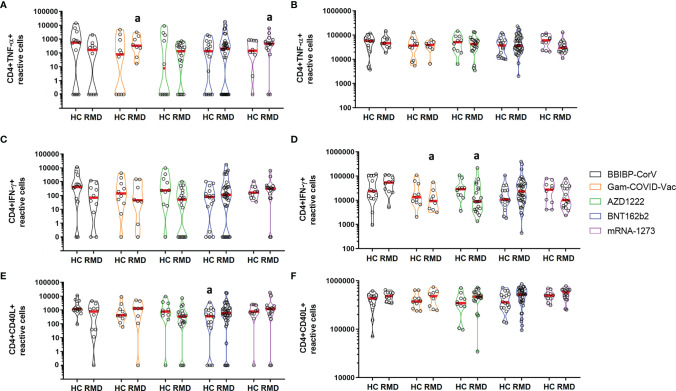
CD4^+^ T-cell responses in healthy controls and RMD patients receiving different types of vaccines upon S/M/N peptide **(A, C, E)** or polyclonal **(B, D, F)** stimulation. HC, healthy controls; RMD, rheumatic and musculoskeletal disease. **(A)** a vs. BBIBP-CorV (RMD) (p = 0.0316). **(D)** a vs. BBIBP-CorV (RMD) (p = 0.0047). **(E)** a vs. BNT162b2 (RMD) (p = 0.011).

Patients who were vaccinated with the BBIBP-CorV vaccine had a lower proportion of TNF-α-producing CD4^+^ cells upon stimulation with S/M/N-peptides compared to those who received the Gam-COVID-Vac or mRNA-1273 vaccines (**Figure 4A**, p = 0.0316), whereas there was no difference upon polyclonal stimulation (**Figure 4B**). Patients, who received the inactivated virus vaccine or vector vaccines showed a tendency for lower proportions of CD4^+^ IFNγ^+^ T-cells compared to healthy controls upon stimulation with S/M/N-peptides (**Figure 4C**). On the other hand, patients who received the BBIBP-CorV vaccine had a higher proportion of IFNγ-producing CD4^+^ cells upon polyclonal stimulation compared to those vaccinated with the Gam-COVID-Vac or AZD1222 vaccines (**Figure 4D**, p = 0.0047). Healthy controls who were vaccinated with the BNT162b2 vaccine had a lower proportion of CD40L^+^ CD4^+^ cells upon stimulation with S/M/N-peptides compared to those who received the BBIBP-CorV vaccine (**Figure 4E**, p = 0.011), whereas there was no difference upon polyclonal stimulation (**Figure 4F**).

The proportion of CD4^+^ TNF-α^+^ responders amongst healthy controls versus patients 4 months post vaccination were 62% vs 20% for BBIBP-CorV, 36% vs 25% for pooled cases of Gam-COVID-Vac and AZD1222, and 31% vs 42% for pooled cases of BNT162b2 and mRNA-1273 vaccinated individuals. The proportion of CD4^+^ IFNγ^+^ responders among healthy controls versus patients 4 months post vaccination were 62% vs 20% for BBIBP-CorV, 36% vs 18% for pooled cases of Gam-COVID-Vac and AZD1222, and 23% vs 28% for pooled cases of BNT162b2 and mRNA-1273 vaccinated individuals. The percentage of CD4^+^ CD40L^+^ responders amongst healthy controls versus patients 4 months post vaccination were 92% vs 60% for BBIBP-CorV, 68% vs 54% for pooled cases of Gam-COVID-Vac and AZD1222, and 62% vs 70% for pooled cases of BNT162b2 and mRNA-1273 vaccinated individuals.

## Discussion

Our study provides detailed information regarding the impact of autoimmune and inflammatory RMDs, disease duration, the immune profile of the patients and various immunosuppressive treatments on vaccine-induced immunogenicity against SARS-CoV-2. We report the results of the first single center prospective study conducted during the COVID-19 pandemic to investigate the complex short and medium-term immune response to a large variety of SARS-CoV-2 vaccines in autoimmune and inflammatory RMDs patients. We demonstrated that all five investigated vaccines were immunogenic in the majority of patients and healthy controls with variable antibody and T-cell response and an acceptable safety profile (**Supplementary Tables 1, 2**). These findings support the results of recent studies where considerable immunogenicity was induced by anti-SARS-CoV-2 mRNA vaccines (26).

SARS-CoV-2 vaccination of RMD patients, however, raises several questions, including how disease activity and immune-suppressive therapy may influence their response. Immunosuppressive drugs may reduce immunogenicity of vaccines, but the protective benefit is generally favorable. The current approach to COVID-19 vaccination of RMD patients is based on data extrapolated from studies on other vaccines, and SARS-CoV-2 vaccination data are scarce. Reduced immunogenicity to anti-SARS-CoV-2 mRNA vaccine by GC therapy was also reported (11, 26). However, there are limitations to drawing a clear conclusion including the low dosage of GC and its combination with other cDMARDs and bDMARDs. Our results confirmed that long term stable low dosage of GC therapy, cDMARDs and most of the bDMARDs have no significant effect on the production of anti-RBD binding antibodies in autoimmune and inflammatory RMDs patients with low disease activity or in remission. The Siemens Advia Centaur sCOVG assay was used in our laboratory to detect the SARS-CoV-2 specific anti-RBD binding antibodies. Irsara et al. showed good correlation (r = 0.84) of Centaur results with virus neutralization titers and concluded that quantitative sCOVG SARS-CoV-2 S1-RBD IgG levels could be used as a surrogate for virus neutralization capacity (27). On the other hand, patients on B-cell inhibitory treatment showed lower antibody responses both at one- and four-months post vaccination compared to those receiving other immune suppressive treatment. The timing of B-cell depleting therapy appears to be a crucial question in terms of vaccination. In case of RMD patients in remission or with a low disease activity, delaying B-cell depleting therapy could potentially prevent the impaired immunogenicity to COVID-19 vaccination. Our results are in line with literature data, that specific antibody production following vaccination may be compromised in patients with B-cell depleting therapy (28).

The risk of impaired immunogenicity based on disease duration in RMD patients with low disease activity has not been documented. The initial antibody response, measured at one month, is higher in patients with shorter disease duration. On the contrary, a longer disease duration even with low disease activity or remission is associated with impaired antibody response, which may be associated with a longer cumulative exposure to immune suppressive therapy. Several studies suggested dynamic changes of the immune phenotype of patients over time in spite of low disease activity or remission (29). Our results suggest that all patients with a long disease duration (over 10 years) require increased attention irrespective of disease activity based on the potential higher risk of reduced immunogenicity. Furthermore, since the antibody response at four months was higher in spondylarthropathies compared to RA and autoimmune RMDs, a disease specific vaccination strategy of RMD patients should also be considered.

It is tempting to speculate whether there is an additional risk of reduced immunogenicity in autoimmune and inflammatory RMDs patients with a higher disease activity. Further studies are needed in this group the patient belongs to. The question of booster vaccine doses of at risk patient groups is extremely important. Recent data confirmed that a homologous additional BNT162b2 vaccine dose and temporary discontinuation of DMARD therapy results in a significant anti-S1 response in the majority of patients with RA who had an impaired anti-S1 response to the standard two-dose vaccination regimen (30). Studies with larger patient cohorts will help determine whether and how often booster vaccination is necessary to optimize the vaccine-induced humoral and cellular immune response in specific patient groups with autoimmune and inflammatory RMDs.

Patients with a positive immune-serological profile, namely, antinuclear antibody, anti-mutated citrullinated vimentin, rheumatoid factor, or anti-neutrophil cytoplasmic antibody show a more rapid decline in anti-RBD neutralizing antibodies by four months post vaccination than patients with a negative immune-serological profile. Both B-cells and T-cells have important roles in the pathogenesis of autoimmune and inflammatory RMDs. However, the proportion of different T-cell subtypes and the amount of autoantibody producing B-cells is variable. Recent studies suggest that RA leads to higher inflammatory activity in seronegative compared with seropositive patients at the time of diagnosis, and the treatment response was lower in seronegative compared with seropositive patients. The highest disease burden of all phenotypes was also found among RA autoantibody-negative patients, at baseline and after 2 years (31). Different therapeutic response was described for new immunomodulatory therapies based on ANA-positivity. This observation possibly relates to the important role of ANAs in cytokine production and tissue inflammation and damage. Several patients with a positive ANA test result were found to have a higher risk of infections. ANA testing was also used as an initial screen in patients with non-specific clinical symptoms, such as fever, joint pain, myalgias, fatigue, rash, or anemia, and the likelihood of an ANA-positive result due to infection was higher (32). The study of Simon et al. measured SARS-CoV-2 specific antibody levels and reported a more heterogeneous composition of patients with immune mediated inflammatory disease (IMID): spondyloarthritis (SpA/psoriatic arthritis) (32.1%), rheumatoid arthritis (RA) (29.8%), inflammatory bowel disease (9.5%), psoriasis (9.5%) and systemic IMIDs (33). Kempis et al. reported a focused study on 53 RA patients versus 20 healthy controls followed by mRNA-based vaccines (9 subjects got mRNA-1273, the rest got BNT162b2) measuring also anti-SARS-CoV-2 specific antibodies (34). Mrak et al. showed that 39% of Rituximab treated IMID patients showed seroconversion followed by mRNA-based vaccination (28). To the best of our knowledge, this is the first study which confirms the role of the immune-serological profile, both cellular and humoral immunity of autoimmune and inflammatory RMDs patients testing 5 different SARS-CoV-2 specific vaccines.

The BBIBP-CorV vaccine appears to produce the lowest, while BNT162b2 and mRNA-1273 vaccines the highest antibody response in both healthy individuals and patients both at one- and four months post immunization. Therefore, using an mRNA-based vaccine in patients receiving B-cell inhibitory therapy is of key importance in view of our finding that these patients showed lower antibody responses both at one and four months in comparison to those receiving other biologicals. Patients vaccinated with the BBIBP-CorV vaccine seem to produce a lower level of humoral response, but a higher cellular response is seen compared to other vaccines upon SARS-CoV-2-specific S/M/N-peptide stimulation. This is in line with the more complex antigenic composition of the inactivated vaccine. The rate of responders was in line with literature data regarding healthy controls (35). Our current results demonstrated lower proportions of autoimmune and inflammatory RMD patients above the diagnostic cut-off value for neutralizing antibody production in comparison to healthy controls, mainly in the cases of inactivated and adenovirus vector-based vaccines. However, T-cell response rates were comparable in autoimmune and inflammatory RMDs patients and controls.

In summary, we confirmed novel clinical risk factors of impaired immunogenicity in autoimmune and inflammatory RMDs patients, and we described the variation of their humoral and cellular immune response to five different SARS-CoV-2 vaccines. This knowledge can influence clinical decision making and inform strategies for vaccination in order to reduce and prevent the occurrence of COVID-19 infections among these patients. Our data draws attention to the importance of booster SARS-CoV-2 vaccines among autoimmune and inflammatory RMDs patients with specific clinical risk factors, namely, disease duration and immune serological profile, and those with RA or autoimmune RMDs. The variable humoral and cellular immune response between the different vaccines might help optimize the timing and dosage of booster vaccination. The differences in humoral and cellular immunogenicity in autoimmune and inflammatory RMDs patients compared to controls may necessitate the implementation of alterations in their vaccination strategy. It may be advantageous to recommend mRNA-based vaccines for patients with RMD, or at least that mRNA-based vaccines are used for booster doses. Overall, there was no hospitalization or death due to COVID-19 in the medical history of the studied RMDs or healthy controls.

## Data Availability Statement

The raw data supporting the conclusions of this article will be made available by the authors, without undue reservation.

## Ethics Statement

The studies involving human participants were reviewed and approved by the Human Investigation Review Board of the University of Szeged. The patients/participants provided their written informed consent to participate in this study.

## Author Contributions

GJS and AB conceived of and designed the study, participated in data collection and analysis. GT and AB supervised clinical data management. AB organized and supervised the vaccination protocol. NG, ES, PN, and JB performed experiments measuring SARS-CoV-2 specific T-cell responses. LIN performed the measurement of SARS-CoV-2 specific neutralizing anti-RBD specific antibodies. DH and AB collected epidemiological and clinical data and assisted with the identification of SARS-CoV-2 infection and follow-up of patients. GJS, GT and AB analyzed the data. GJS and GT prepared the figures. GJS and LGP were responsible for laboratories. GJS verified the data and had access to raw data. GJS, GT, and AB wrote the manuscript, and GJS, ZS, GT, and AB revised the manuscript. GJS and AB had final responsibility for the decision to submit for publication. All authors helped to edit the manuscript. All authors listed have made a substantial, direct, and intellectual contribution to the work and approved it for publication.

## Funding

This study was supported by the grant 2020-1.1.6-JÖVŐ-2021-00003 from the National Research, Development, and Innovation Office (NKFIH), Hungary. This manuscript was prepared with the professional support of the doctoral student scholarship program of the Ministry of Innovation and Technology, financed by the National Research, Development, and Innovation Fund for JB (KDP-17-4/PALY-2021, 1000464).

## Conflict of Interest

Author GS was employed by the company Cs-Smartlab Devices Ltd. Authors LN and LP were employed by the company Avidin Ltd.

The remaining authors declare that the research was conducted in the absence of any commercial or financial relationships that could be construed as a potential conflict of interest.

## Publisher’s Note

All claims expressed in this article are solely those of the authors and do not necessarily represent those of their affiliated organizations, or those of the publisher, the editors and the reviewers. Any product that may be evaluated in this article, or claim that may be made by its manufacturer, is not guaranteed or endorsed by the publisher.
