# Supplementary material for: Humoral and Cellular Immunogenicity and Safety of Five Different SARS-CoV-2 Vaccines in Patients With Autoimmune Rheumatic and Musculoskeletal Diseases in Remission or With Low Disease Activity and in Healthy Controls: A Single Center Study
Source: Front Immunol. 2022 Mar 31;13:846248. doi: 10.3389/fimmu.2022.846248 (PMC9008200; doi:10.3389/fimmu.2022.846248)
Supplement: Supplementary file 1 [file Presentation_1.pptx]

## Slide 1
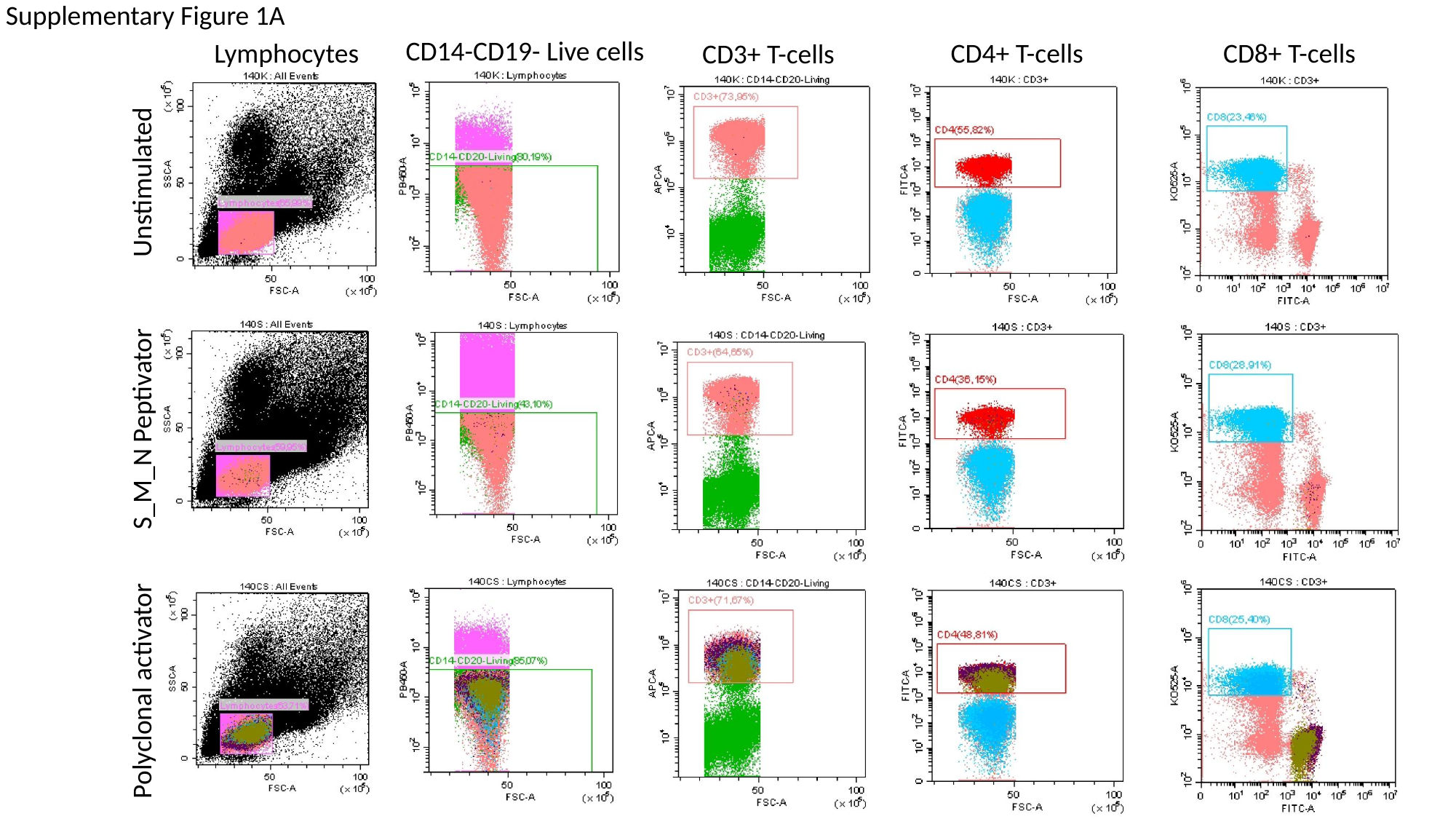

Supplementary Figure 1A
CD14-CD19- Live cells
Lymphocytes
CD4+ T-cells
CD8+ T-cells
CD3+ T-cells
Unstimulated
S_M_N Peptivator
Polyclonal activator

## Slide 2
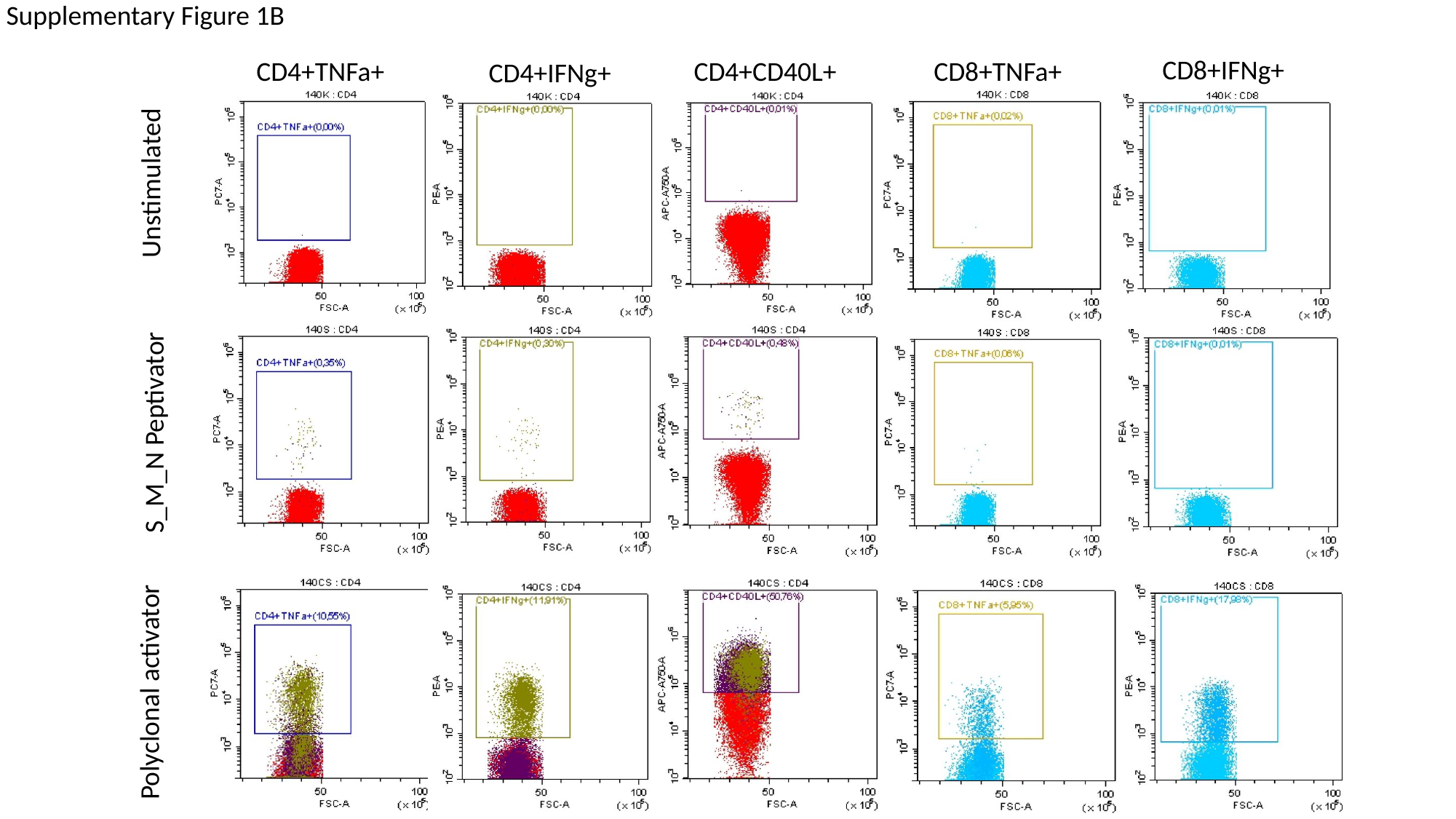

Supplementary Figure 1B
CD8+IFNg+
CD8+TNFa+
CD4+TNFa+
CD4+CD40L+
CD4+IFNg+
Unstimulated
S_M_N Peptivator
Polyclonal activator
