# Supplementary material for: Humoral and Cellular Immunogenicity and Safety of Five Different SARS-CoV-2 Vaccines in Patients With Autoimmune Rheumatic and Musculoskeletal Diseases in Remission or With Low Disease Activity and in Healthy Controls: A Single Center Study
Source: Front Immunol. 2022 Mar 31;13:846248. doi: 10.3389/fimmu.2022.846248 (PMC9008200; doi:10.3389/fimmu.2022.846248)
Supplement: Supplementary file 2 [file Table_1.docx]

**Supplementary Table 1. Adverse events after the first dose of the five investigated vaccines in healthy controls and RMD patients**

|  | BNT162b2 | | Gam-Covid-Vac | | BBIBP-CorV | | mRNA-1273 | | AZD1222 | |
| --- | --- | --- | --- | --- | --- | --- | --- | --- | --- | --- |
|  | HC  n=16 | RMD  n = 34 | HC  n=16 | RMD  n = 8 | HC  n=18 | RMD  n = 11 | HC  n=10 | RMD  n = 16 | HC  n=14 | RMD  n = 20 |
| Local reactions | | | | | | | | | | |
| Pain | 9 | 4 | 8 | 1 | 7 | 2 | 5 | 6 | 9 | 2 |
| Erythema | 3 | 2 | 1 | 0 | 0 | 2 | 4 | 2 | 1 | 2 |
| Swelling | 5 | 0 | 3 | 0 | 2 | 0 | 6 | 2 | 1 | 2 |
| Pruritus | 3 | 1 | 0 | 0 | 1 | 0 | 2 | 2 | 0 | 0 |
| Tingling | 2 | 0 | 1 | 0 | 2 | 0 | 0 | 0 | 1 | 0 |
| Systemic reactions | | | | | | | | | | |
| Fever | 0 | 1 | 6 | 0 | 1 | 0 | 3 | 1 | 8 | 1 |
| Nausea | 0 | 1 | 0 | 0 | 0 | 1 | 0 | 0 | 0 | 0 |
| Vomiting | 0 | 0 | 0 | 0 | 0 | 0 | 0 | 0 | 0 | 0 |
| Rhinorrhea | 0 | 1 | 0 | 0 | 0 | 0 | 0 | 0 | 0 | 1 |
| Cough | 0 | 1 | 0 | 0 | 0 | 0 | 0 | 0 | 0 | 2 |
| Myalgia | 8 | 6 | 4 | 1 | 0 | 0 | 3 | 3 | 5 | 1 |
| Arthralgia | 1 | 4 | 2 | 1 | 1 | 1 | 0 | 2 | 2 | 1 |
| Chills | 2 | 3 | 5 | 0 | 0 | 0 | 3 | 1 | 8 | 2 |
| Malaise | 2 | 5 | 9 | 1 | 2 | 0 | 5 | 3 | 9 | 3 |
| Headache | 1 | 2 | 4 | 0 | 0 | 0 | 0 | 1 | 6 | 1 |
| Allergic reaction | 0 | 1 | 0 | 0 | 0 | 0 | 0 | 0 | 0 | 0 |
| Dizziness | 0 | 0 | 2 | 0 | 0 | 1 | 0 | 1 | 1 | 0 |
| Throat pain | 0 | 0 | 1 | 0 | 0 | 0 | 2 | 0 | 1 | 0 |
| Chest pain/palpitations | 0 | 0 | 1 | 0 | 0 | 0 | 0 | 0 | 0 | 0 |
| Diarrhoea | 0 | 0 | 0 | 0 | 0 | 1 | 0 | 0 | 0 | 0 |
| Pruritus | 0 | 0 | 0 | 0 | 0 | 0 | 0 | 0 | 0 | 0 |
| Lack of appetite | 0 | 0 | 0 | 0 | 0 | 0 | 0 | 0 | 1 | 0 |
| Local lymphadenopathy | 1 | 0 | 0 | 0 | 0 | 0 | 3 | 1 | 0 | 0 |
| High blood pressure | 0 | 0 | 0 | 0 | 0 | 0 | 0 | 0 | 0 | 0 |
| Uveitis | 0 | 0 | 0 | 0 | 0 | 0 | 0 | 0 | 0 | 0 |
| Herpes zoster | 0 | 0 | 0 | 0 | 0 | 0 | 0 | 0 | 0 | 0 |
| Pericarditis | 0 | 0 | 0 | 0 | 0 | 0 | 0 | 0 | 0 | 0 |
| Vaginal bleeding | 0 | 0 | 0 | 0 | 0 | 0 | 0 | 0 | 0 | 0 |

The total number of mild or moderate local and systemic reactions in healthy controls (HC) and RMD patients was 189 and 87, respectively. No serious adverse events were observed.

**Supplementary Table 2. Adverse events after the second dose of the five investigated vaccines in healthy controls and RMD patients**

|  | BNT162b2 | | Gam-Covid-Vac | | BBIBP-CorV | | mRNA-1273 | | AZD1222 | |
| --- | --- | --- | --- | --- | --- | --- | --- | --- | --- | --- |
|  | HC  n=16 | RMD  n = 34 | HC  n=16 | RMD  n = 8 | HC  n=18 | RMD  n = 11 | HC  n=10 | RMD  n = 16 | HC  n=14 | RMD  n = 20 |
| Local reactions | | | | | | | | | | |
| Pain | 8 | 3 | 7 | 1 | 6 | 1 | 5 | 6 | 6 | 0 |
| Erythema | 1 | 1 | 2 | 0 | 0 | 1 | 4 | 0 | 0 | 1 |
| Swelling | 3 | 1 | 5 | 0 | 0 | 0 | 5 | 3 | 1 | 1 |
| Pruritus | 1 | 1 | 0 | 0 | 0 | 0 | 1 | 2 | 0 | 0 |
| Tingling | 2 | 0 | 1 | 0 | 1 | 1 | 0 | 0 | 0 | 0 |
| Systemic reactions | | | | | | | | | | |
| Fever | 3 | 0 | 3 | 0 | 1 | 0 | 3 | 3 | 1 | 1 |
| Nausea | 0 | 1 | 0 | 0 | 0 | 1 | 1 | 0 | 0 | 0 |
| Vomiting | 0 | 0 | 0 | 0 | 0 | 0 | 1 | 0 | 0 | 0 |
| Rhinorrhea | 0 | 0 | 0 | 0 | 0 | 0 | 0 | 0 | 0 | 2 |
| Cough | 0 | 1 | 0 | 0 | 0 | 0 | 0 | 0 | 0 | 1 |
| Myalgia | 7 | 3 | 5 | 1 | 1 | 0 | 3 | 1 | 2 | 0 |
| Arthralgia | 4 | 3 | 2 | 1 | 1 | 1 | 1 | 1 | 1 | 2 |
| Chills | 6 | 2 | 3 | 0 | 2 | 0 | 5 | 2 | 2 | 0 |
| Malaise | 6 | 2 | 8 | 1 | 5 | 0 | 5 | 4 | 4 | 2 |
| Headache | 3 | 1 | 3 | 0 | 2 | 0 | 1 | 3 | 2 | 0 |
| Allergic reaction | 0 | 0 | 0 | 0 | 0 | 0 | 0 | 0 | 0 | 0 |
| Dizziness | 2 | 2 | 2 | 0 | 1 | 1 | 0 | 0 | 0 | 0 |
| Throat pain | 0 | 0 | 1 | 0 | 0 | 0 | 2 | 0 | 0 | 0 |
| Chest pain/palpitations | 0 | 0 | 0 | 0 | 0 | 0 | 0 | 0 | 0 | 0 |
| Diarrhoea | 0 | 0 | 0 | 0 | 1 | 1 | 0 | 0 | 0 | 0 |
| Pruritus | 0 | 0 | 0 | 0 | 0 | 0 | 0 | 0 | 0 | 0 |
| Lack of appetite | 0 | 0 | 0 | 0 | 0 | 0 | 0 | 0 | 0 | 0 |
| Local lymphadenopathy | 1 | 0 | 0 | 0 | 0 | 0 | 2 | 0 | 0 | 0 |
| High blood pressure | 0 | 0 | 0 | 0 | 0 | 0 | 0 | 0 | 0 | 0 |
| Uveitis | 0 | 0 | 0 | 0 | 0 | 0 | 0 | 1 | 0 | 0 |
| Herpes zoster | 0 | 0 | 0 | 0 | 0 | 0 | 0 | 0 | 0 | 0 |
| Pericarditis | 0 | 0 | 0 | 0 | 0 | 0 | 0 | 0 | 0 | 0 |
| Vaginal bleeding | 0 | 0 | 0 | 0 | 0 | 0 | 0 | 1 | 0 | 0 |

The total number of mild or moderate local and systemic reactions in healthy controls (HC) and RMD patients was 168 and 69, respectively. No serious adverse events were observed.
